# Supplementary material for: Multiscale mechanisms of green concrete regulated by silicon-to-calcium ratio: Physico-mechanical properties, hydration structure, and durability performance
Source: PLoS One. 2026 Apr 28;21(4):e0348144. doi: 10.1371/journal.pone.0348144 (PMC13124005; doi:10.1371/journal.pone.0348144)
Supplement: Attachment — Submitted filename: Response to Reviewers.doc [file pone.0348144.s002.doc]

#### Response to the Editor and Reviewer Comments

**The manuscript number is** 9PONE-D-26-04987

**Title: Multiscale mechanisms of green concrete regulated by silicon-to-calcium ratio: Physico-mechanical properties, hydration structure, and durability performance**

**PLOS One**

**Author(s):** Jiayuan Lou, Yiwen Liang, Wenhua Zha, Qiang Su

Dear Editor, Dear reviewers

We highly appreciate your and the reviewers’ comments and suggestions, which are very helpful for improving our paper. Based on these comments and suggestions, we have made careful modifications on the manuscript. Appended to this letter is our point-to-point response to the comments raised by the reviewers. These comments were reproduced and our responses to each reviewer’s comments were given.

We hope that all these changes fulfill the requirements to make the manuscript acceptable for publication in the journal of **PLOS One.** If you have any questions, please contact us immediately. We are grateful for your attention to our manuscript. Once again, thanks very much for your arduous work and instructive suggestions to our manuscript processing.

Yours Sincerely,

Jiayuan Lou, Yiwen Liang, Wenhua Zha, Qiang Su

Correspondence author: Yiwen Liang ;E-mail:38102@qzc.edu.cn ; ---------------------------------------------------------------

College of Civil Engineering and Architecture

Quzhou University

Quzhou 324000，China

**Reviewer #1:**

Thank you for your invitation to review the manuscript entitled “Multiscale mechanisms of green concrete regulated by silicon-to-calcium ratio: Physico-mechanical properties, hydration structure, and durability performance” for the journal. The manuscript presents a good and meaningful piece of work and contributes useful information regarding the utilization of silicon-rich solid wastes in green concrete systems; however, the paper still requires minor revision before it can be considered for publication.

With respect and many thanks to the editor, the following minor comments are provided for the authors’ consideration.

**Q1. Although the study emphasizes the regulation effect of the Si/Ca ratio on the mechanical and durability performance of WGRC, the manuscript does not clearly explain the calculation procedure and compositional derivation of the Si/Ca ratio in the mixture design stage, therefore the authors should explicitly describe how the ratio was quantified considering the chemical contributions of glass sand, glass powder, rice husk ash, and cement phases.**

**Author's Response:** Thank you for your valuable suggestions. While a brief description of the Si/Ca ratio calculation was included in the original manuscript, we acknowledge that the procedure was not sufficiently explicit. In the revised manuscript, we have clarified the calculation method by explicitly introducing the molar-based formulation of the Si/Ca ratio (SiO_2_/CaO) and providing the corresponding equation. In addition, we have clearly defined the contributions of each component in the mixture: cement was considered as the primary source of CaO, while waste glass powder and rice husk ash were treated as the main contributors of reactive SiO_2_.**Line 122~130:** The Si/Ca ratio was calculated based on the molar ratio of SiO_2_ to CaO derived from the oxide compositions of the raw materials listed in Table 1. Specifically, the molar amounts of SiO_2_ and CaO contributed by each binder component were determined by dividing their mass fractions by the corresponding molar masses. Referring to the chemical compositions in Table 1, the intrinsic Si/Ca ratios of cement, GP, and RHA were calculated as 0.304, 5.54, and 76.8, respectively. In this study, the dosage ratio of GP to RHA was fixed at 1:1 to maintain a balanced contribution of silica sources and to control variables in the mixture design, and their total contents were adjusted to achieve the target Si/Ca ratios. The specific mix proportions and corresponding Si/Ca ratios are presented in Table 2.

**Table 2. Mix ratio (Unit: kg/m^3^)**

| Sample | Silica-calcium ratio | Cement | Fine aggregate | Coarse aggregate | Glass sand | Glass powder | Rice husk ash | Water |
| --- | --- | --- | --- | --- | --- | --- | --- | --- |
| S/C-0.34 | 0.34 | 357.06 | 443.1 | 1202 | 189.9 | 5.47 | 5.47 | 208 |
| S/C-0.40 | 0.40 | 346.12 | 443.1 | 1202 | 189.9 | 10.94 | 10.94 | 208 |
| S/C-0.46 | 0.46 | 335.18 | 443.1 | 1202 | 189.9 | 16.41 | 16.41 | 208 |
| S/C-0.52 | 0.52 | 324.24 | 443.1 | 1202 | 189.9 | 21.88 | 21.88 | 208 |
| S/C-0.58 | 0.58 | 313.30 | 443.1 | 1202 | 189.9 | 27.35 | 27.35 | 208 |
| S/C-0.64 | 0.64 | 302.36 | 443.1 | 1202 | 189.9 | 32.82 | 32.82 | 208 |
| S/C-0.70 | 0.70 | 291.42 | 443.1 | 1202 | 189.9 | 38.29 | 38.29 | 208 |
| S/C-0.76 | 0.76 | 280.48 | 443.1 | 1202 | 189.9 | 43.76 | 43.76 | 208 |

**Q2. The microstructural characterization used to explain the hydration mechanisms (such as SEM, XRD, or related analysis) should be more quantitatively interpreted, because the current discussion mainly relies on qualitative observations and does not sufficiently correlate the evolution of C-S-H structure and gel composition with the measured macroscopic mechanical properties and durability indicators.**

**Authors’ response**: Thank you for your valuable suggestions. We have re-examined the microstructural analysis in the manuscript. In the original version, the EDS results were not fully utilized, and the relationship between gel composition and macroscopic properties was not clearly described.

The manuscript has been revised as follows: In Section 3.5, the EDS results in Table 6 are further analyzed, and the variation of the Si/Ca ratio in C-S-H gel with mixture proportion and curing age is described with specific values;In addition, the microstructural interpretations in this paper are primarily based on SEM and EDS results and should be considered semi-quantitative analyses.**Line 342 and 354：**From Table 6, it can be observed that the regulation of the calcium-silicon ratio significantly affects the calcium-silicon ratio in the internal C-S-H gel of WGRC at different curing ages. Based on the atomic percentages of Si and Ca, the Si/Ca ratio of the gel phase was calculated, and the results are summarized in Table 6. It can be observed that the gel-phase Si/Ca ratio increased consistently with both the designed mixture Si/Ca ratio and curing age. For example, at 7 days, the gel-phase Si/Ca ratio increased from 1.22 for the 0.34 mixture to 1.72 for the 0.76 mixture, while at 90 days it further increased to 1.36 and 1.82, respectively. This indicates that the incorporation of glass powder and rice husk ash continuously promoted the enrichment of silicate species in the hydration products during curing.

However, the increase in gel-phase Si/Ca ratio did not lead to a monotonic improvement in macroscopic performance. Instead, the compressive strength, splitting tensile strength, flexural strength, and shear strength generally exhibited optimal values within a moderate mixture Si/Ca range, rather than at the highest Si/Ca level. This suggests that the role of Si/Ca regulation is not merely to increase the silicon content of the gel, but to optimize the balance between gel polymerization, reaction continuity, and calcium supply .

**Table 6. Silica-calcium ratio of C-S-H gel in WGRC**

| Silica-calcium ratio | Curing age | | |
| --- | --- | --- | --- |
|  | 7d | 28d | 90d |
| 0.34 | 1.22 | 1.30 | 1.36 |
| 0.40 | 1.27 | 1.33 | 1.39 |
| 0.46 | 1.35 | 1.38 | 1.45 |
| 0.52 | 1.41 | 1.47 | 1.52 |
| 0.58 | 1.50 | 1.55 | 1.60 |
| 0.64 | 1.58 | 1.62 | 1.67 |
| 0.70 | 1.63 | 1.71 | 1.75 |
| 0.76 | 1.72 | 1.78 | 1.82 |

**Q3. In the durability evaluation part, especially for water penetration resistance, sulfate attack, and carbonation behavior, the manuscript should provide better descriptions of the testing standards, boundary conditions, and specimen preparation procedures…**

**Authors’ response**: Thank you for reviewing this article carefully. We have made changes in the revised manuscript. The testing methods, boundary conditions, and specimen preparation procedures are described in Section 2.3, and the adopted testing standards (ASTM C1202) are stated in Section 6.1.

**Q4. The discussion related to the optimal Si/Ca ratio range (0.46~0.58) should be further strengthened by presenting a more rigorous parametric comparison of mechanical strength, durability indices, and microstructural evolution within this interval… literature can also be further enhanced, Frontiers in Materials, vol. 11. DOI: 10.3389/fmats.2024.1392875 ;;; Innovative Infrastructure Solutions, Springer, vol. 9. DOI: 10.1007/s41062-024-01571-w ;;;; Construction and Building Materials, Elsevier, vol. 472. DOI: 10.1016/j.conbuildmat.2025.140960**

**Authors’ response**: Thank you very much for your valuable suggestions. The references suggested by the reviewer are relevant to the effect of the Si/Ca ratio on material performance and have been cited in the revised manuscript. Additional discussion has also been included to compare the mechanical properties, durability indicators, and microstructural characteristics within the selected Si/Ca range, in order to clarify the rationale for the proposed optimal range.

**Q5. In several sections of the results and discussion, the linkage between macro-scale performance (strength and durability) and multi-scale hydration structure evolution is mentioned but not fully demonstrated…**

**Authors’ response**: We sincerely thank the reviewers for their insightful comments. Although the relationship between macroscopic properties and hydration structures was discussed in the original manuscript, the relevant description was not sufficiently clear. To address this, we have provided additional explanations in the relevant discussion section to further elaborate on the connection between mechanical properties, durability, and the evolution of hydration structures.

**Line 618 and 621：**It should be noted that the present microstructural interpretation is primarily based on EDS-derived gel composition and SEM morphology; therefore, the discussion is semi-quantitative in nature. More advanced quantitative techniques such as XRD Rietveld refinement, TGA, or MIP may be employed in future work to further verify phase evolution and pore structure changes.

**Reviewer #2:**

**Q1. The study investigates Si/Ca ratios ranging from 0.34 to 0.76. It would be helpful if the authors explain the rationale behind selecting this specific range and increment interval. Were these values based on previous research, preliminary tests, or theoretical considerations?**

**Authors’ response**: We sincerely thank the reviewers for their constructive comments, which are crucial for enhancing the reproducibility and rigor of this study. The selected Si/Ca ratio range (0.34~0.76) was determined based on commonly reported ranges in previous studies, combined with the feasible mixture design using glass powder and rice husk ash in this study. This range covers relatively low to high Si/Ca conditions to capture the overall variation trend in material performance. The increment between adjacent levels was chosen to ensure sufficient resolution for trend analysis while keeping the experimental workload manageable.

**Line 122~130:** The Si/Ca ratio was calculated based on the molar ratio of SiO_2_ to CaO derived from the oxide compositions of the raw materials listed in Table 1. Specifically, the molar amounts of SiO_2_ and CaO contributed by each binder component were determined by dividing their mass fractions by the corresponding molar masses. Referring to the chemical compositions in Table 1, the intrinsic Si/Ca ratios of cement, GP, and RHA were calculated as 0.304, 5.54, and 76.8, respectively. In this study, the dosage ratio of GP to RHA was fixed at 1:1 to maintain a balanced contribution of silica sources and to control variables in the mixture design, and their total contents were adjusted to achieve the target Si/Ca ratios. The specific mix proportions and corresponding Si/Ca ratios are presented in Table 2.

**Table 2. Mix ratio (Unit: kg/m^3^)**

| Sample | Silica-calcium ratio | Cement | Fine aggregate | Coarse aggregate | Glass sand | Glass powder | Rice husk ash | Water |
| --- | --- | --- | --- | --- | --- | --- | --- | --- |
| S/C-0.34 | 0.34 | 357.06 | 443.1 | 1202 | 189.9 | 5.47 | 5.47 | 208 |
| S/C-0.40 | 0.40 | 346.12 | 443.1 | 1202 | 189.9 | 10.94 | 10.94 | 208 |
| S/C-0.46 | 0.46 | 335.18 | 443.1 | 1202 | 189.9 | 16.41 | 16.41 | 208 |
| S/C-0.52 | 0.52 | 324.24 | 443.1 | 1202 | 189.9 | 21.88 | 21.88 | 208 |
| S/C-0.58 | 0.58 | 313.30 | 443.1 | 1202 | 189.9 | 27.35 | 27.35 | 208 |
| S/C-0.64 | 0.64 | 302.36 | 443.1 | 1202 | 189.9 | 32.82 | 32.82 | 208 |
| S/C-0.70 | 0.70 | 291.42 | 443.1 | 1202 | 189.9 | 38.29 | 38.29 | 208 |
| S/C-0.76 | 0.76 | 280.48 | 443.1 | 1202 | 189.9 | 43.76 | 43.76 | 208 |

**Q2. The mix design fixes the waste glass powder to rice husk ash ratio at 1:1. The manuscript should justify this assumption and discuss whether other ratios were considered. Exploring different proportions might reveal more optimized combinations for performance improvement.**

**Authors’ response**: We sincerely appreciate your insightful comments, which are essential to enhancing the reliability and persuasiveness of the findings in this study. In this study, the ratio of waste glass powder to rice husk ash was fixed at 1:1 to control variables in the mixture design, allowing the effect of the Si/Ca ratio on material performance to be the primary focus. In addition, waste glass powder and rice husk ash provide different forms of silica in the system, and the 1:1 ratio was selected as a balanced combination to represent their combined effect. The influence of different proportions of waste glass powder and rice husk ash is indeed of interest, but it is beyond the scope of the present study and may be considered in future work.**Q3. The study appears to lack a conventional concrete control mix without solid waste materials. Including such a control sample would allow clearer comparison and better evaluation of the real benefits of the proposed waste-based concrete system.**

**Authors’ response**: We sincerely appreciate the reviewer's insightful comments. This study investigates the effect of the Si/Ca ratio within a waste-based cementitious system, and the comparison is carried out among mixtures with different Si/Ca ratios. All mixtures were designed within the same material system, and the analysis is based on the variation of mechanical and durability performance with different Si/Ca ratios. Including a conventional concrete control mix would allow further comparison, but it is beyond the scope of this study and may be considered in future work.**Q4. The results show that slump decreases significantly with increasing Si/Ca ratio. However, the manuscript does not discuss whether the observed workability remains acceptable for practical construction applications. The authors should comment on practical workability requirements.**

**Authors’ response**: We sincerely appreciate your constructive suggestions, which provide valuable guidance for improving the readability of the article. In the original manuscript, the variation of slump with the Si/Ca ratio was analyzed, but its practical applicability was not discussed. In the revised manuscript, the specific slump range (75~195 mm) has been added, and it is clarified that all values remain within the workable range for normal concrete, indicating their applicability in practical construction. The relevant clarification has been included in the slump analysis section.**Line 176~200:** The slump of waste glass and rice husk ash concrete (WGRC) with different silicon-calcium ratios is shown in Table 3. It was observed that as the silicon-calcium ratio increased, the slump of WGRC decreased. This may be because cement, glass powder, and rice husk ash formed other calcium hydration products during the mixing process. The microstructure of these products may increase the viscosity of the cement paste, thus reducing the flowability of the concrete [35]. It should be noted that the decrease in slump of WGRC exhibited a staged characteristic: when the silicon-calcium ratio increased from 0.34 to 0.58, the slump decreased from 195 mm to 150 mm, a reduction of 45 mm. On average, for every 0.06 increase in the silicon-calcium ratio, the slump decreased by about 10~15 mm; when the silicon-calcium ratio increased from 0.58 to 0.76, the slump decreased from 150 mm to 75 mm, a total reduction of 75 mm. On average, for every 0.06 increase in the silicon-calcium ratio, the slump decreased by about 22~28 mm. This is primarily because, when the silicon-calcium ratio is less than 0.58, the incorporation of glass powder and rice husk ash mainly serves to fill microvoids, optimizing the paste structure and reducing the confinement of free water, which allows the flowability to remain at a relatively high level.

However, when the silicon-calcium ratio exceeds 0.58, the SiO_2_ content significantly increases, promoting the formation of a large amount of C-S-H gel, which quickly thickens the paste, thus exacerbating the loss of flowability and leading to a sharp decrease in slump [36]. Additionally, a higher amount of glass powder and rice husk ash enhances the interaction forces between particles, hindering the flow of the paste and causing a rapid slump reduction. Although the slump decreased with increasing silicon-calcium ratio, the measured values ranged from 75 mm to 195 mm, which remain within the workable range for normal concrete applications.

**Fig 6. Slump fitting results**

Based on the data in Table 3, the relationship between the slump of WGRC and the silicon-calcium ratio was fitted, and the fitting result shows a power function decreasing trend. The fitting results are shown in Fig 6. The fitted results in Fig 6 can be referenced to understand the slump variation trend of WGRC.

**Table 3. Slump test results**

| Silica-calcium ratio | 0.34 | 0.40 | 0.46 | 0.52 | 0.58 | 0.64 | 0.70 | 0.76 |
| --- | --- | --- | --- | --- | --- | --- | --- | --- |
| Slump/mm | 195 | 188 | 178 | 165 | 150 | 128 | 100 | 75 |
| Reduction range/% | _ | 3.59 | 8.72 | 15.38 | 23.08 | 34.36 | 48.72 | 61.54 |

**Q5. It is suggested to add article entitled “Saeed et al. Evaluating the Efficiency of Alkaline Activator with Silica-Rich Wastes in Stabilizing Cadmium-Contaminated Soil” to the literature review.**

**Authors’ response**: We sincerely appreciate the reviewer's insightful and valuable comments. The recommended reference has been cited in the revised manuscript.**Line 691~693:** [23] Saeed K, Al-Khyat S, Hacheem Z A, et al. Evaluating the efficiency of alkaline activator with silica-rich wastes in stabilizing cadmium-contaminated soil. Civil Engineering Journal, 2024, 10(7): 2123-2143. https://doi.org/10.28991/CEJ-2024-010-07-04

**Q6. The paper reports that water absorption increases with increasing Si/Ca ratio, which contradicts some previous studies. Although the authors attribute this to the water absorption capacity of rice husk ash, further microstructural evidence or pore structure analysis would strengthen this explanation.**

**Authors’ response:** We sincerely appreciate your valuable suggestions, which are essential for improving the readability of the paper. In the original manuscript, the increase in water absorption was mainly attributed to the water absorption capacity of rice husk ash, but the explanation was not sufficiently detailed. In the revised manuscript, additional clarification has been included, indicating that rice husk ash has a porous structure and high specific surface area, which can increase water absorption capacity and contribute to the observed trend. Further microstructural or pore structure analysis will be a key focus of future work.

**Q7. The results indicate that early strength decreases with higher Si/Ca ratios but later strength improves at moderate ratios. The authors should provide deeper discussion on the hydration kinetics and gel formation mechanisms responsible for this transition.**

**Authors’ response**: We sincerely appreciate the reviewer's insightful and valuable comments. These suggestions provide important guidance for enhancing the reliability and engineering applicability of our study's conclusions. In the original manuscript, the explanation for the reduction in early strength and the improvement in later-age strength at moderate Si/Ca ratios was not sufficiently detailed. In response, additional discussion has been added in the revised manuscript. It is clarified that the lower early strength at higher Si/Ca ratios is mainly related to the slower early reaction of glass powder and rice husk ash and the reduced relative calcium availability in the system. With increasing curing age, the active SiO2 gradually participates in the pozzolanic reaction and forms additional C-S-H gel, which contributes to later-age strength development. It is also clarified that when the Si/Ca ratio is excessively high, the available calcium becomes insufficient to sustain a more effective continuous gel network, and therefore the later-age strength does not continue to increase. As a result, moderate Si/Ca ratios show better later-age performance. For a detailed analysis, see Section 3.3**Line 233~318:** The compressive strength test results of WGRC are shown in Fig 8. It is observed that at 7 days, the compressive strength decreases with the increase in silicon-calcium ratio. When the silicon-calcium ratio increases from 0.34 to 0.76, the compressive strength decreases from 23.02 MPa to 12.35 MPa. This indicates that at early ages, a lower silicon-calcium ratio has a higher hydration rate and better early strength development. This trend is similar to the reports by Wei et al. [40] and Hot et al. [44]. This is mainly because the higher Ca content promotes the rapid formation of C-S-H gel, enhancing the compactness of the early structure. Furthermore, with the increase in silicon-calcium ratio, the decrease in Ca content may reduce the formation rate of C-S-H gel, thereby affecting the early strength gain. A too high silicon-calcium ratio may result in insufficient framework support, leading to a decrease in structural stability and, consequently, a decrease in compressive strength.

**Fig 8. Compressive strength**

At 28 days, the compressive strength still exhibits a similar trend, with low silicon-calcium ratio samples showing relatively higher compressive strength. For example, when the silicon-calcium ratio is 0.34, the compressive strength is 31.07 MPa. Additionally, compared to the 7-day age, the compressive strength of WGRC increases, and the compressive strength of samples with a silicon-calcium ratio between 0.46 and 0.58 approaches 30 MPa, indicating that this range may be more suitable for the silicon-calcium ratio. It is worth noting that for high silicon-calcium ratios (0.7 and above), the compressive strength is still relatively low, which may be due to the reduced stability of the gel structure and higher porosity [45].

At 90 days, the compressive strength of WGRC generally further increases, reaching the maximum value at a silicon-calcium ratio of 0.58. This indicates that a moderate silicon-calcium ratio can promote the stable growth of C-S-H gel, improving long-term strength. It should be noted that as the silicon-calcium ratio increases further, the compressive strength decreases. For example, when the silicon-calcium ratio is 0.76, the compressive strength is only 25.5 MPa. This may be due to the higher Si content leading to the gel structure being dominated by silicate phases, reducing its hardening performance, and the pore structure being more open, resulting in a decrease in overall compactness [46,47]. Furthermore, Hu et al. [48] also pointed out that insufficient Ca content affects the crosslinking degree of C-S-H gel, leading to a decrease in compressive strength.

The splitting tensile strength test results of WGRC are shown in Fig 9. As seen in Figure 9, at 7 days, the splitting tensile strength of WGRC decreases gradually with the increase in silicon-calcium ratio, which is similar to the trend observed for compressive strength. This may be due to the fact that at early ages, samples with a lower silicon-calcium ratio have a higher calcium content, which promotes the rapid formation of C-S-H gel and enhances the bonding strength of the interfacial transition zone, thus improving tensile strength [49]. On the other hand, samples with a higher silicon-calcium ratio have insufficient calcium content, leading to slower growth of C-S-H gel, which results in lower splitting tensile strength.

**Fig 9. Splitting tensile strength**

At 28 days, the trend in splitting tensile strength differs from that at 7 days, with a peak value appearing at a silicon-calcium ratio of 0.52. This suggests that, at this stage, the growth and structure of C-S-H gel have been optimized, leading to better splitting tensile strength. Additionally, for low silicon-calcium ratio samples, the excessive calcium content may lead to insufficient crosslinking of C-S-H gel, resulting in a decrease in the compactness of the microstructure [50]. When the silicon-calcium ratio exceeds 0.58, the splitting tensile strength decreases significantly, indicating that a higher silicon-calcium ratio may weaken the interfacial transition zone, thereby reducing tensile strength.

At 90 days, the peak value of splitting tensile strength occurs at a silicon-calcium ratio of 0.58, slightly delayed compared to 28 days. This suggests that as the curing age increases, the splitting tensile strength of samples with a high silicon-calcium ratio gradually surpasses that of low silicon-calcium ratio samples due to the continuous development of C-S-H gel. It should be noted that when the silicon-calcium ratio exceeds 0.58, the splitting tensile strength gradually decreases. This phenomenon is similar to the results reported by Chen et al. [51]. Chen et al. [51] suggested that this might be due to insufficient calcium content in high silicon-calcium ratio samples during long-term curing, which reduces the crosslinking capacity of C-S-H gel, making it difficult to effectively repair microcracks, thus affecting splitting tensile strength. It is important to emphasize that this study further extended the diversity of silicon elements by incorporating glass powder and rice husk ash, causing the optimal range of silicon-calcium ratio to shift to the right.

The bending strength test results of WGRC are shown in Fig 10. As observed, at 7 days, the bending strength gradually decreases with the increase in silicon-calcium ratio. This trend suggests that at early curing stages, samples with lower silicon-calcium ratios have a higher concentration of calcium ions, which promotes faster formation of early C-S-H gel and a denser interfacial transition zone, thus enhancing the bending strength of the concrete. However, with the increase in silicon-calcium ratio, the decrease in calcium content leads to a slower early formation rate of C-S-H gel, resulting in a more porous interfacial transition zone, which affects the bending strength.

At 28 days, the trend of bending strength changes, with the peak value occurring at a silicon-calcium ratio of 0.46. Compared to 7 days, the bending strength of WGRC increases, but the growth rate varies with different silicon-calcium ratios. The bending strength of lower silicon-calcium ratio samples (0.34 and 0.4) remains relatively high (4.47 MPa and 4.68 MPa, respectively), but the increase is smaller compared to samples with a silicon-calcium ratio of 0.46. This is because, at lower silicon-calcium ratios, C-S-H gel has already formed to a sufficient extent during the early stage, resulting in a more gradual growth in the later stage, whereas at a silicon-calcium ratio of 0.46, the C-S-H gel continues to develop at 28 days, improving the interfacial transition zone and leading to the maximum bending strength.

**Fig 10. Flexural strength**

At 90 days, the peak bending strength is further delayed, occurring at a silicon-calcium ratio of 0.52. As the curing age increases, the bending strength of samples with silicon-calcium ratios of 0.46 and 0.52 improves due to the continued growth of C-S-H gel and the further enhancement of the interfacial transition zone. However, when the silicon-calcium ratio exceeds 0.58, the bending strength decreases significantly (e.g., 4.05 MPa at a silicon-calcium ratio of 0.76). This may be because, during long-term curing, samples with higher silicon-calcium ratios have lower calcium content, leading to insufficient growth of C-S-H gel, resulting in a more porous microstructure that affects the bending capacity of the concrete .

The reduction in early-age strength at higher Si/Ca ratios is mainly related to the slower reaction of glass powder and rice husk ash compared with cement hydration. At early ages, the increase in Si/Ca ratio reduces the relative calcium availability in the system, while the silica-rich supplementary materials have not yet reacted sufficiently to form enough binding products. As a result, the formation of strength-contributing hydration products is limited, leading to lower early strength. With increasing curing age, the pozzolanic activity of glass powder and rice husk ash becomes more evident. The active SiO_2_ gradually reacts with Ca(OH)_2_ to form additional C-S-H gel, which improves matrix compactness and enhances later-age strength. However, when the Si/Ca ratio becomes excessively high, the available calcium in the system is insufficient to sustain the formation of a more effective continuous gel network. Therefore, later-age strength is improved at moderate Si/Ca ratios rather than increasing continuously with increasing Si/Ca ratio.

**Q8. The use of gray relational analysis to evaluate mechanical performance is interesting. However, the manuscript should explain why this method was selected instead of more commonly used statistical approaches such as regression analysis or multi-criteria decision analysis.**

**Authors’ response**: We sincerely appreciate the reviewer's highly constructive insights, which provide crucial guidance for enhancing the engineering applicability of this research. Gray relational analysis was adopted in this study because it is suitable for multi-indicator evaluation with limited sample size and does not require strict assumptions on data distribution. In this study, multiple mechanical performance indicators were involved, and the number of samples was relatively limited. Gray relational analysis allows for a direct comparison among different indicators, and therefore it was selected.

**Q9. It is also suggested to add articles entitled “Yudhistira et al. Optimizing Concrete Mix Design for Cost and Carbon Reduction Using Machine Learning” and “Ulloa et al. Optimizing Waste Foundry Sand in Concrete Considering Strength Properties for Sustainable Green Structures” to the literature review.**

**Authors’ response**: We sincerely appreciate the reviewer's insightful and valuable comments. The recommended reference has been cited in the revised manuscript.

**Q10. SEM and EDS analyses are used to explain the changes in hydration products. Nevertheless, the manuscript would benefit from more detailed quantitative analysis of microstructure, such as pore size distribution or phase quantification, to better support the proposed mechanisms.**

**Authors’ response**: We sincerely appreciate the reviewer's insightful and valuable comments. We acknowledge that the microstructural analysis in the manuscript does not provide more detailed quantitative results. In this study, SEM and EDS were used to analyze hydration products, while quantitative analysis such as pore size distribution or phase quantification was not included. It has been clarified in the revised manuscript that the present microstructural analysis is mainly based on SEM and EDS and is therefore semi-quantitative. More detailed analysis of pore structure and phase composition would be valuable, and this will be considered in future work.

**Q11. The variable-angle shear test provides valuable insights into structural behavior. However, the choice of shear angles (25°, 45°, and 65°) should be justified. The authors should explain whether these angles correspond to specific engineering applications or theoretical considerations.**

**Authors’ response**: We sincerely appreciate the reviewer's insightful and valuable comments. The rationale for selecting the shear angles was not clearly stated in the original manuscript. In the revised manuscript, it has been clarified that the selected angles (25°, 45°, and 65°) represent low, intermediate, and high inclination conditions, and that 45° is a commonly adopted reference angle in shear-related studies for comparison of mechanical behavior.

**Line 148~152:** (4) Variable-angle shear tests were conducted at 28 days of curing. The shear angles were set at 25°, 45°, and 65°, respectively. The selected shear angles (25°, 45°, and 65°) were chosen to represent low, intermediate, and high inclination conditions, with 45° corresponding to a commonly adopted reference angle in shear-related studies. The specimens were cubic in shape with dimensions of 50 mm×50 mm×50 mm. The tests were carried out under displacement-controlled loading at a constant rate of 0.5 mm/min until specimen failure.
